# Bridging gaps in perinatal mental health services for socioeconomically disadvantaged women

**DOI:** 10.3389/fgwh.2026.1850520

**Published:** 2026-09-23

**Authors:** Lem Ngongalah Udeozor, Catherine El-Zerbi, Josephine Wildman, Malcolm Moffat, Judith Rankin

**Affiliations:** 1 Population Health Science Institute, Newcastle University, Newcastle-upon-Tyne, United Kingdom; 2 Division of Health Research, Faculty of Health & Medicine, Lancaster University, Lancaster, United Kingdom; 3 IDEAS-NET, Northumbria University, Newcastle-upon-Tyne, United Kingdom

**Keywords:** health inequalities, healthcare access, maternal mental health services, perinatal mental health, socioeconomic disadvantage

## Abstract

**Introduction:**

Perinatal mental health (PMH) is a critical aspect of maternal and child wellbeing, yet many women, particularly those from socioeconomically disadvantaged backgrounds, face significant barriers in accessing timely and appropriate support. While national policies have aimed to improve PMH services, disparities in awareness, accessibility, and engagement persist. This study explored the challenges and facilitators to PMH service access in the North East and North Cumbria (NENC) region in England, with a focus on identifying actions to improve accessibility.

**Methods:**

A two-phase qualitative study was conducted with 43 participants. Phase 1 involved semi-structured interviews with service providers from healthcare, voluntary, and community sectors who support women experiencing socioeconomic disadvantage (*n* = 23). Phase 2 comprised interviews with women from diverse, socioeconomically disadvantaged backgrounds who had experienced PMH challenges (*n* = 20). Data were analysed thematically and subsequently mapped to Levesque's conceptual framework of healthcare access, to examine individual and system-level factors influencing access to and engagement with PMH services.

**Results:**

Findings highlight multiple barriers to accessing PMH care, including the lack of routine mental health discussions in antenatal care, limited visibility of services, cultural stigma, and structural challenges such as service fragmentation and system distrust. Women from disadvantaged backgrounds often faced additional barriers due to digital exclusion, language difficulties, and lack of childcare. Participants identified six key actions to improve access: embedding PMH discussions into routine maternity care, enhancing service visibility through targeted outreach, expanding flexible service models, strengthening cultural competence, building trust through community-based models, and improving coordination and referral pathways between PMH support and existing healthcare and social services.

**Discussion:**

Addressing barriers to PMH access requires a multi-level approach that prioritises proactive service integration, workforce capacity building, and community-led engagement strategies. Strengthening service coordination and ensuring culturally responsive and flexible care models are critical steps toward improving equity in PMH support.

## 1 Introduction

Perinatal mental health (PMH) encompasses the emotional, psychological and social wellbeing of women during pregnancy and the first year after childbirth. Access to PMH support is essential for the timely identification and management of mental health challenges during the perinatal period (1). This period is a critical window for maternal and child health, as untreated PMH conditions, such as antenatal and postnatal depression, anxiety disorders, postpartum psychosis and post-traumatic stress disorder, can increase the risk of persistent maternal mental illness and impaired functioning, and adversely affect the child's emotional, behavioural and cognitive development (2). Early intervention is essential to prevent long-term adverse outcomes and reduce progression to more severe mental health conditions.

PMH has become an increasing policy and clinical priority in England, with substantial investment and service development in recent years. However, access to PMH services remains uneven, and many women struggle to access timely support, particularly those who are marginalised or living in socioeconomically disadvantaged circumstances (3, 4). Various national initiatives have been introduced to strengthen PMH provision and improve access to care, with progress partly driven by findings from MBRRACE-UK's Confidential Enquiries into Maternal Deaths, particularly in relation to maternal suicide, alongside advocacy from women with lived experience (5). MBRRACE-UK has also highlighted persistent ethnic inequalities in maternal mortality and morbidity, with disproportionately poor maternal outcomes among Black women and some other minority ethnic groups (5). The Maternal Mental Health Alliance (MMHA) has played a key role in expanding services (6), while PMH care pathways have supported integration by setting out processes for identifying, assessing, referring and treating women across maternity, primary care and specialist mental health services (7). The NHS Maternity Transformation Programme and wider policy commitments, including the NHS Long Term Plan and Mental Health Implementation Plan, contributed to the expansion of specialist PMH provision across England (8–10). This included the development of specialist community and inpatient services, such as Mother and Baby Units and Maternal Mental Health Services, to support women with more complex needs. This policy focus has continued under the 10 Year Health Plan for England (11). The push for better PMH care has also been strengthened by the National Health Service (NHS)'s commitment to parity of esteem, which recognises that mental health should be given the same priority as physical health (12).

Despite this expansion, services are not consistently accessible or responsive to women's diverse clinical, cultural, linguistic and practical needs. Women from socioeconomically disadvantaged backgrounds continue to face multiple barriers, including geographical, cultural, linguistic, financial, and structural constraints (4, 13–15). In addition, some women's needs do not align with existing service models, and provision is not always tailored to diverse communities (16, 17). Evidence from the MMHA highlights substantial variation in service provision, access criteria, and waiting times across England, indicating ongoing inequities in access to care (18). Addressing these inequities remains a national priority. The Women's Health Strategy for England emphasises the need for more accessible and responsive maternal mental health support (19). Improving equitable access to PMH services also aligns with wider government priorities on moving from hospital-centred care to community-based provision, and from treatment-focused models towards preventive care (11).

Improving access to PMH services is especially important in North East and North Cumbria (NENC), where high levels of deprivation and geographical variation may compound barriers to accessing services (20).

This qualitative study aimed to identify barriers and facilitators to accessing PMH services among women experiencing socioeconomic disadvantage in the North East and North Cumbria (NENC), and actions to improve access. The study included the perspectives of service providers supporting socioeconomically disadvantaged women and women with lived experience of PMH difficulties.

## 2 Materials and methods

### 2.1 Study design and setting

This was a two-phase qualitative study involving semi-structured interviews with: (i) service providers from diverse backgrounds, including healthcare, voluntary organisations, charities, community services, and social enterprises; and (ii) women from socioeconomically disadvantaged backgrounds in the NENC who had experienced PMH challenges. The study was conducted across North East and North Cumbria, a region of approximately 3.2 million people (20). The region encompasses major urban centres, coastal communities and rural areas (21). Around 32% of the population live in neighbourhoods within the 20% most deprived areas in England (20). More than 26,000 babies are born in the region each year, indicating a substantial population potentially requiring perinatal healthcare and support (22).

### 2.2 Sampling and recruitment

Participants in phase 1 were purposively recruited based on their professional experience of working with women from socioeconomically disadvantaged backgrounds in the NENC. These included: primary care clinical staff practising in areas of deprivation, community pharmacy staff, non-clinical staff allied to medicine (e.g., link workers, community health workers), voluntary and community sector workers, and social workers. Recruitment efforts also sought to include service providers working with groups often underrepresented in maternal health research, including migrant women, women seeking asylum, women from ethnic minority communities, women experiencing homelessness, women with insecure access to healthcare or wider social support, and non-binary and transgender people who had experienced giving birth and faced PMH challenges.

Recruitment was conducted through a flyer distributed via email, WhatsApp, Facebook and Twitter, detailing study information, eligibility criteria, and participation instructions. Additional participants were identified through direct outreach to relevant organisations via professional networks and directories. Snowball sampling was also used, allowing participants to share the study invitation within their networks.

Participants in the second phase were also recruited purposively, leveraging the professional networks established in phase 1. Phase 2 inclusion criteria were: (1) living in NENC; (2) experience of mental health difficulties during pregnancy or within the first year after childbirth; and (3) experience of socioeconomic disadvantage. Participants were eligible regardless of English proficiency, and interpretation support was available if needed. Service providers were asked to share the study invitation with the women they supported, with a focus on recruiting participants from socioeconomically disadvantaged groups. These included women from lower-income households (e.g., those receiving means-tested benefits such as Universal Credit or housing support or living in areas of high deprivation as defined by the Index of Multiple Deprivation), as well as single mothers, women from ethnic minority backgrounds, women living with disabilities, and women with experiences of domestic or sexual abuse. Recruitment efforts were further supplemented through flyers shared on social media platforms (Facebook, Twitter, and community forums), and participant referrals (snowballing). Recruitment continued across the two phases until no substantially new themes were identified.

### 2.3 Data collection

Interviews were conducted virtually through Microsoft Teams or Zoom depending on participants' preference, between September 2021 and January 2023. Interviews with service providers explored disadvantaged populations in healthcare, types of PMH and wider mental health support available to women living in deprivation, approaches for identifying and assessing PMH distress, referral pathways, and barriers (including the impact of COVID-19), and facilitators to access PMH services. Interviews with women explored perceptions of perinatal mental illness, interpretations of mental health in general, ways of identifying PMH distress, and challenges related to finding, accessing and engaging with PMH services. Interviews were audio-recorded and transcribed using the automated transcription function in Microsoft Teams or Zoom, with transcripts checked against the recordings for accuracy. Written informed consent was obtained from all participants before the interviews. Ethical approval was obtained from Newcastle University for Phase 1 (Ref: 13509/2020), and from NHS REC for Phase 2 (IRAS ID 304315).

### 2.4 Patient and public involvement

Five women of child-bearing age who had experienced maternity care and PMH support in the UK contributed to the design and methodology of this research. The women were invited through a mother-baby group in the North East of England, and the discussions were held virtually as they took place during COVID-19. Findings from these discussions informed the development of the study topic guide for phase 2.

### 2.5 Data analysis

The interview data were analysed thematically to explore participants' experiences and perspectives. Codes were developed inductively from the interview data and grouped into patterns from which themes were developed. Themes were identified based on recurring patterns in the data and their relevance to the study aim. Three researchers (LN, CE, JW) coded the same two transcripts and compared their coding, with differences discussed to refine the coding and emerging themes. One researcher (LN) then coded the remaining transcripts, with themes discussed and refined through repeated discussions with the wider research team. The resulting themes were subsequently mapped to Levesque's conceptual framework of healthcare access, with the mapping reviewed and discussed by the research team to examine barriers and facilitators at individual and structural levels (23). This framework includes five key dimensions: approachability (awareness of services), acceptability (alignment with cultural and social norms), availability/accommodation (access in terms of time and location), affordability (financial accessibility), and appropriateness (service relevance and quality). These dimensions influence individuals' ability to recognise the need for care, seek and reach services, afford treatment, and engage with providers. Affordability did not emerge as a distinct recurrent theme in the final analysis. While some service providers in phase 1 identified cost as a potential barrier for women from deprived backgrounds, the women themselves did not report cost as a reason for being unable to access PMH services. This may reflect that PMH services in England are largely delivered through the NHS and are free at the point of use, although indirect costs (e.g., travel, time off work, or childcare) are known to influence access (24). Service provider interviews were used to identify areas for improving access to PMH services, while women's accounts were used to contextualise the barriers these areas sought to address.

### 2.6 Presentation of findings

The results section focuses on actions that can improve access to PMH services, drawing on insights from service providers and the experiences of women from socioeconomically disadvantaged communities. Findings are supported by direct quotes, providing context to the challenges faced, strategies identified by providers to enhance accessibility, and examples of measures implemented to address these issues. Participants are assigned unique identifiers (e.g., P1, P2) to maintain anonymity. Quotes from mothers are labelled with their participant number followed by “Mum” in brackets (e.g., P1, mum). Quotes from service providers are labelled with their participant number and professional role, with healthcare professionals generally denoted as HCP (e.g., P1, HCP). However, when a specific role is particularly relevant to the context of a quote, it is explicitly stated (e.g., P3, Midwife; P5, Charity Worker).

## 3 Results

### 3.1 Study participants

A total of 43 participants took part in the study, 23 service providers and 20 women with lived experience of PMH challenges.

The 23 service providers represented a multidisciplinary group of professionals from healthcare, local authority, social care, charity, and community settings. They included 10 healthcare professionals (midwives, health visitors, GPs, mental health practitioners, and therapists); five local authority and social care staff (asylum support officers and social workers); and eight charity, voluntary sector, and community-based workers (community workers, peer support workers, support practitioners, mother-and-baby group leads, and baby bank workers). Service providers worked across statutory and non-statutory services and had experience supporting women with antenatal and postnatal care, maternal mental health, safeguarding, housing and welfare needs, parenting support, refugee and asylum-related issues, community integration, and referral or signposting to wider services. Many worked closely with women facing multiple and intersecting disadvantages, including poverty, housing insecurity, migration-related challenges, language barriers, trauma, domestic abuse, social isolation, and difficulties accessing mainstream maternity and mental health services. Their roles ranged from frontline support to service leadership, management, and coordination.

Twenty women aged 19–41 years took part in the study. All the women had experienced mental health difficulties during pregnancy or within the first year after childbirth, and their experiences included both accessing and attempting to access PMH support. Participants included married (*n* = 9), divorced (*n* = 4), and single women (*n* = 7). Most were mothers of young children, with 12 caring for a baby under 1 year of age. Descriptive characteristics of the phase 2 participants are presented in Supplementary Table 1. The sample was ethnically diverse and included women experiencing multiple and overlapping forms of socioeconomic disadvantage including childcare pressures, limited social support, wider caring responsibilities, language barriers, unfamiliarity with the UK healthcare system, domestic or sexual abuse, disability, bereavement following baby loss, and culturally shaped barriers to help-seeking. Three women participated with interpretation support.

### 3.2 Disadvantaged and under-represented groups in healthcare access

Service providers identified a wide range of disadvantaged and under-represented groups who faced significant barriers to healthcare access. These included women living on low incomes, those experiencing rural disadvantage or insecure living conditions such as homelessness or inadequate housing, survivors of sexual or domestic abuse, women recovering from substance use disorders, women with physical disabilities, single and younger mothers, migrants, asylum seekers, refugees, women from ethnic minority communities, mothers with multiple or adopted babies or disabled children, sex workers, and lesbian, bisexual, trans, and queer people with the capacity to give birth. Participants also emphasised the importance of intersectionality, highlighting that many women experience multiple, overlapping forms of disadvantage, which compounds barriers and further restricts their access to essential healthcare services.

Service providers identified six key areas for improving access to PMH services: 1) integrating mental health discussions into routine antenatal care; 2) enhancing service visibility through targeted outreach, signposting, and accessible information; 3) expanding flexible service delivery to accommodate diverse needs; 4) embedding cultural sensitivity in service design to ensure inclusivity and responsiveness; 5) strengthening community engagement to build trust and encourage uptake; 6) and integrating PMH support within existing services frequently accessed by women, such as primary care, social services, and community support programmes. Figure 1 shows how the key barriers and facilitators across the findings map onto Levesque's framework. These themes are explored further below, incorporating perspectives from both service providers and women with lived experience.

**Figure 1:**
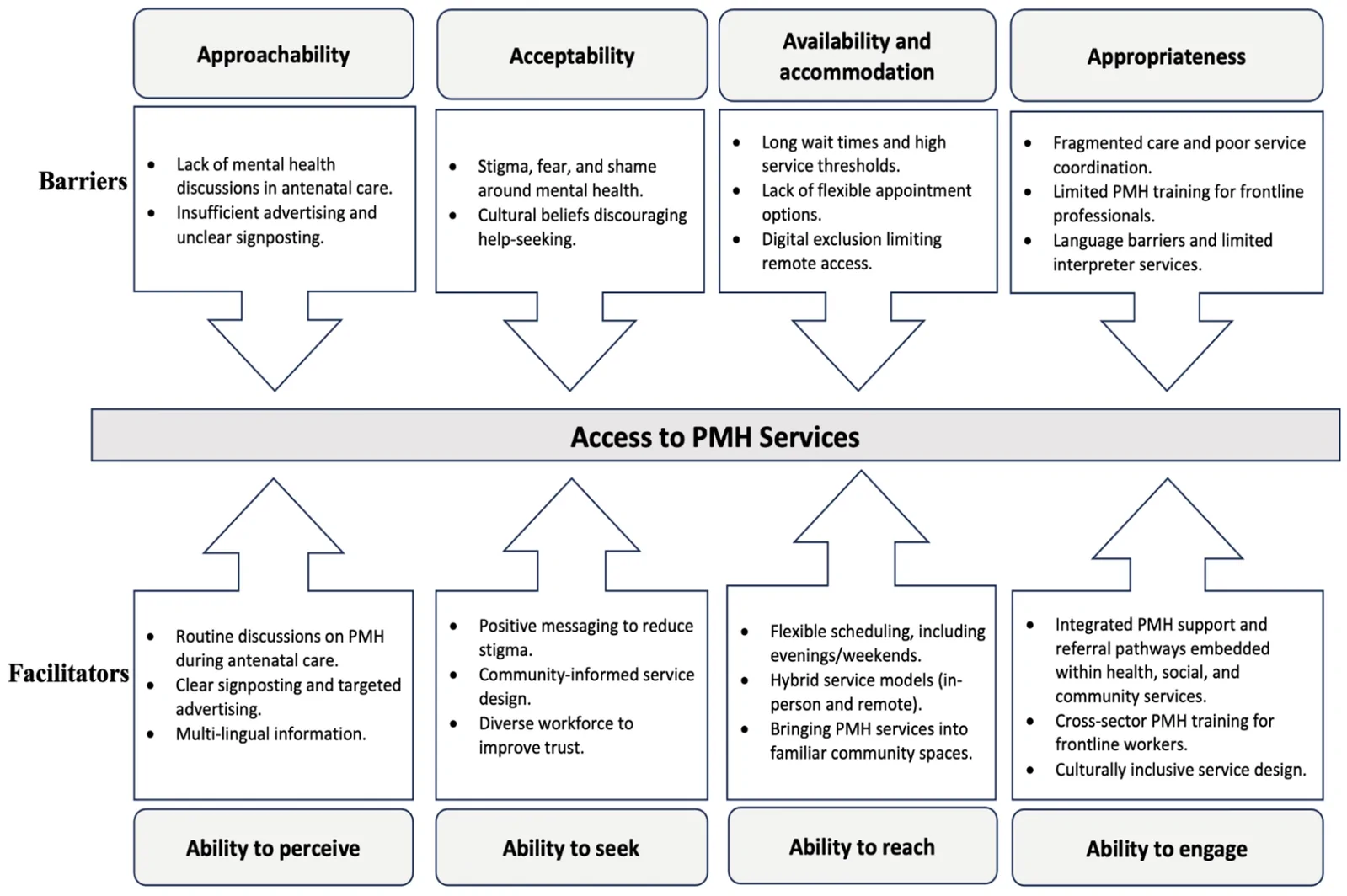
Key barriers and facilitators to accessing PMH services, mapped onto Levesque's framework for healthcare access. Figure 1 was adapted from Levesque et al.'s conceptual framework of access to healthcare (23), licensed under CC BY 2.0, and modified by the authors for the purposes of this study.

#### 3.2.1 Embedding mental health discussions into routine antenatal care

Participants identified a significant gap in routine antenatal care, where discussions about physical health dominated, while mental health was often overlooked. Midwives and other healthcare professionals acknowledged that while routine check-ups focused on monitoring the mother's and baby's physical wellbeing, conversations about emotional and psychological health were infrequent or absent:

“We tend to focus more on the physical health of the mother and the baby during pregnancy, don't we? But mental health is just as important, and we need to be advocates for these conversations … and to ensure they're happening regularly, so yes, as midwives, we need to be starting these conversations with our patients.” (P14, Midwife)

This lack of discussion was seen as a missed opportunity to normalise mental health conversations and equip women with the knowledge to recognise the symptoms of perinatal mental illness, which many women are often unaware of:

“…a lot of women aren't very aware of it, in terms of the risks or the signs of getting mentally ill, or even the risk it might pose to their baby as well. These conversations happen a lot less than they should.” (P11, HCP)

Women with lived experience of PMH challenges described feeling unsupported and uninformed, with little guidance on what to expect or where to seek help. As such, many struggled to distinguish between typical emotional and psychological changes that can occur during pregnancy and after birth, and potential signs of perinatal mental illness:

“They don't tell you what to expect, what you might experience, or even how to know whether it could be a mental problem or not. It's not easy to just know that what I'm feeling or what I'm going through has got to do with my mental health. But then, nobody tells you what it could be like or how you can get help.” (P28, Mum)

Even when mental health was mentioned during antenatal appointments, some women found the discussions too brief and lacking in practical guidance:

“She did say something at one of my scans, I think, but it was just barely mentioned, like yeah, the baby blues, you may feel depressed or whatever, but it was just that. There wasn't any real discussion, and nothing about who you can call or whatever if you need help.” (P26, Mum)

Service providers pointed out that women cannot seek help for something they do not fully understand, especially as mental health difficulties during the perinatal period can manifest in ways that may not be immediately recognisable, particularly for first-time mothers. Service providers emphasised the need for mental health discussions to be a standard part of antenatal care, ensuring that women are informed, supported, and directed to available resources before reaching a crisis point. A charity worker highlighted the importance of introducing these conversations early, as many parents later trace their anxiety back to challenges they faced before birth:

“I think it should be started in pregnancy. When you're having things like your antenatal classes and stuff, I think it should be talked about a lot more then. When you do speak to a lot of new parents, some of them, they can trace the anxiety back to whether it's been difficult getting pregnant or even just the experience of being pregnant or the birthing. It would be good for them to have some support from the healthcare providers that they talk to during that period, even if it's just signposting them to services that can help them.” (P1, Charity worker)

#### 3.2.2 Improving signposting and information sharing on PMH services

Beyond the lack of mental health discussions in antenatal care, many women and healthcare professionals highlighted the broader issue of limited access to clear, reliable, and accessible information about PMH services. Even when women recognised that they needed help, they often struggled to find practical guidance on where to seek support.

The lack of service visibility often left women feeling lost and overwhelmed when trying to navigate the healthcare system. Many described the frustration of searching for support without clear direction, a problem compounded by insufficient advertising and unclear pathways to care:

“There's not really much advertising around mental health. You see all the big signage saying, “Support your mental health today,” with blah-blah-blah. But where are the places that you can actually go? There's no real advertisement to say, “We are here for you. This is where we are.” I think being accessible means being known about. If you don't know about them, how are you ever going to find them?” (P27, Mum)

Unfamiliarity with the UK healthcare system added another layer of difficulty, particularly for women who were new to the country or had limited knowledge of how healthcare services were organised and accessed. These difficulties were particularly pronounced in communities with limited awareness of available services. For example, the Roma population faced additional barriers due to a lack of information and support in navigating the system:

“If you look at the research or anything around the Roma community, it suggests that they don't access things particularly well. But that's because they either don't really understand the system or how they can access information. We just expect people, when they turn up here, to understand how things work and to just know where to find information they need to help them access services.” (P13, Community health visitor)

The limited visibility of PMH services was also a challenge for healthcare professionals. Several acknowledged that gaps in their knowledge of available PMH services caused uncertainty about what support existed and how to direct women to the right resources:

“We often find ourselves limited by a lack of information when it comes to signposting them to perinatal mental health services. I work in a busy general practice, and we see a lot of pregnant women and new mothers. I don't think we have any specialised perinatal mental health services in our area, and I don't know how to access the ones that are available elsewhere.” (P14, Midwife)

This lack of knowledge among healthcare professionals could leave women having to search for support themselves, adding to the difficulty of seeking help when already experiencing distress. Women from disadvantaged backgrounds could face further challenges where access to information and other resources was limited.

Language barriers also restricted access to information and support. English-only written information could be difficult to use without assistance from family or friends, leaving some women unable to understand important healthcare information:

“Most time we twist and throw in bin, because we don't understood, we don't know what the letter mean, and if you say this is the information that you need to help you in pregnancy, but I can't make sense, what I do for that? Unless maybe I have cousin, or friend, she help me read it and tell me, then I understand.” (P37, Mum)

Women faced additional challenges where interpretation support was unavailable, which made it difficult to communicate their needs or understand healthcare guidance. This was particularly concerning for those in distress who needed immediate support:

“People tell us they've been to a GP appointment and there wasn't an interpreter available, and that's been a barrier. Somebody is already feeling anxious about talking about how depressed and suicidal they are, and then there isn't somebody to even express that in their own language when they're upset and distressed. That's a massive barrier for people.” (P20, Council worker)

Service providers acknowledged that inadequate advertising of PMH services exacerbated healthcare inequalities, particularly for socioeconomically disadvantaged women who already faced barriers due to limited resources and lack of information. To address these gaps, they emphasised the need for targeted outreach and clearer communication. They suggested increasing awareness through campaigns in GP surgeries, maternity clinics, and community centres, ensuring that information about available support is more widely and consistently shared. The importance of multilingual resources was repeatedly highlighted, alongside the need for user-friendly online platforms that provide clear, accessible guidance on service availability and referral pathways.

#### 3.2.3 Expanding access through flexible and hybrid service delivery

Offering PMH support outside traditional working hours, including early mornings, evenings, and weekends, was seen as particularly beneficial for women from disadvantaged backgrounds, many of whom juggled multiple jobs, worked long hours, and lacked affordable childcare or family support:

“Many of the women we work with have young children and are single parents. They have no family support, and no childcare arrangements in place. They have to choose between caring for their children or seeking help for their mental health.” (P10, Charity worker)

This struggle to balance responsibilities made it very difficult for some women to attend appointments, leaving them without access to much-needed support*:*

“I have two young children, and I work full-time as well, so finding the time to attend appointments is just impossible. It's suffocating and sometimes it feels like I'm drowning, but I can't take time off work, and I can't afford to pay for childcare. Got to do my best to handle all that but also means I can't have any time for myself.” (P27, Mum)

To address access barriers, service providers recommended a hybrid approach, offering both in-person and remote options, offering greater flexibility to accommodate women's schedules and individual preferences. While some women preferred face-to-face interactions for emotional support, remote support was seen as particularly beneficial for those who might otherwise struggle to attend in-person appointments due to geographical barriers, such as long distances or limited transportation options. Remote support also provided advantages in terms of privacy and emotional comfort, as the idea of discussing sensitive mental health topics in-person felt overwhelming for some:

“I don't like talking face-to-face especially when it's a difficult subject to talk about. I get very easily triggered, and it's sometimes really difficult trying to talk to someone while also being triggered and trying to remain composed. I feel like if you had that online instead, if you needed to burst into tears, you could, because you're just behind a screen or a phone, that sort of thing.” (P25, Mum)

Service providers reflected that remote service provision was one of the few positive developments in care delivery resulting from COVID-19. While the pandemic posed significant challenges, the shift to online support introduced a level of flexibility that proved particularly beneficial for women with childcare responsibilities, mobility challenges, or difficulties traveling to appointments.

The shift to online access allowed women to access support more easily without the logistical burdens of arranging childcare or navigating transportation challenges. However, service providers also acknowledged that for some women, particularly those from disadvantaged backgrounds or whose first language was not English, virtual provision created new barriers instead of removing them:

“We found that we didn't reach some mams online, especially those who were perhaps most deprived, so those who are sort of at risk of homelessness. That sort of group, because they didn't have the technology or the data. If they had a phone, they couldn't keep it topped up to an extent where they could actually access online.” (P13, Community health visitor)

“We didn't tend to reach people for whom English was a second language just because of the added–I suppose the added barrier of doing things online. Sometimes it can just be so difficult to even get them to download, like, you know, Zoom and these kinds of things. So, access is a real issue. I think it's great when somebody has got the choice and they feel confident about using the technology, but for so many of our clients, access is a real issue.” (P8, Social enterprise worker)

Service providers emphasised the importance of a flexible, hybrid service delivery model that combined both in-person and remote options, allowing women to access support in ways that best fit their circumstances:

“We offer evening and weekend appointments alongside telehealth options to make things easier for them…it means they can talk to someone after the kids are in bed or not have to worry about getting a bus across town. It's about making support fit into their lives, not the other way around” (P12, Charity worker)

#### 3.2.4 Embedding cultural sensitivity in service delivery

Awareness of and responsiveness to people's cultural and religious backgrounds was identified as fundamental to building trust and ensuring meaningful engagement with PMH services. Service providers highlighted that cultural beliefs significantly shape how individuals perceive mental health, their willingness to seek support, and their interactions with healthcare providers. Without an understanding of these factors, service providers risk creating barriers that make mental health services feel inaccessible or unrelatable. One provider highlighted the need for cultural understanding:

“Many women come from backgrounds where mental health is rarely discussed or is seen as a private family matter. If we don't approach this with cultural understanding, they won't feel comfortable engaging with us.” (P16, Social worker)

These cultural influences could affect how women express their need for support, sometimes in ways that did not align with conventional diagnostic expectations. For example, women may describe physical symptoms rather than directly expressing emotional struggles:

“Rather than saying, “Yes, I'm feeling low.” It might be actually, “No, I've got a really bad back, and I've got this, that, and the other.” So, it's doing your assessment around your understanding of that person and their background and where they've come from, and what their understanding of mental health may be.” (P3, Charity worker)

Religious and cultural norms were also identified as factors influencing service accessibility, particularly for women. A community worker shared an example of how cultural and religious factors could impact women's ability to attend sessions and the adjustments required to accommodate them:

“I've worked with some women whose husbands will come in first to check and make sure there are no men on site before they can let their women come in to attend our sessions. This has to happen each time, for every session. So obviously, her ability to come in depends on the man's availability to come in and do those checks before.” (P7, Community worker)

For many women, cultural factors also influenced whether they sought help at all. Some participants described mental distress as either heavily stigmatised or so normalised that professional support was not considered necessary:

“Where I come from, there's nothing like mental health (laughs). Everybody is stressed and depressed, and it's a normal thing that you go through, whether you're pregnant or not. So yes, it's hard for me, but it's not anything new for me to feel like that. It's not something that I'll think, “Oh, maybe I need to ask for help or see a doctor,” because to me, to us, it's normal.” (P29, Mum)

For others, mental illness was associated with extreme psychiatric conditions, contributing to a fear of social exclusion:

“In my culture, people think that if you have a mental health condition, it means your brain is broken or defective in some way. I remember growing up, I was always told that mental illness was something that only happened to “crazy” people. So, nobody wants to say they have a problem with their mental health because there's that fear of being ostracised and people labelling you as mad.” (P28, Mum)

Given these challenges, service providers emphasised the importance of culturally sensitive communication that reframed mental health as an integral part of overall wellbeing rather than something to be feared or hidden. Some highlighted the benefits of recruiting staff from diverse backgrounds, as this was seen to foster trust and improve engagement:

“When women see someone who understands their background, their struggles, and even speaks their language, it completely changes how comfortable they feel about opening up. It makes seeking support feel more welcoming.” (P19, Mental health practitioner)

Cultural competency training was identified as essential for healthcare professionals to build confidence in engaging with diverse communities. A healthcare professional noted that practitioners may hesitate to raise mental health concerns with individuals from different backgrounds, fearing that doing so might be insensitive:

“I suppose we need some training to build the confidence to talk to people who we don't share things with–who are from different cultures and different backgrounds. Health professionals are sometimes more reticent about raising issues with people who are less like themselves. So you might think there could be an issue here, but you leave it alone because you're worried it might be insensitive to ask, unless they choose to talk about it. And so I think that's where some of the health inequality comes from” (P14, HCP).

Training was also seen as crucial for those working with refugee women, many of whom have experienced trauma that affected their mental health in complex ways:

“I think more training could be given to healthcare professionals about women who are from refugee backgrounds and the things that are affecting them, including trauma they will have experienced before. A lot of women might have experienced sexual assault, and being aware of those previous experiences can help pick out signs that they might be struggling, especially with things that they probably won't be able to put in words” (P20, Council worker).

To further support cultural sensitivity in service delivery, some participants suggested integrating creative therapeutic approaches that allow women to express themselves in ways that align with their cultural values and personal comfort. These approaches were seen as particularly valuable for women who may feel uncomfortable with talking therapies or who prefer more hands-on, interactive forms of support. Service providers highlighted that creative therapies could be particularly beneficial in addressing cultural and linguistic barriers, as they allow for expression beyond verbal communication. Visual aids, such as drawings, pictures, and videos, were cited as effective tools for facilitating conversations about mental health, particularly for women whose first language is not English or who were unaccustomed to discussing emotional challenges in a Western medical framework. For women from disadvantaged backgrounds, service providers noted that creative therapeutic approaches that did not use the “mental health” label could be less intimidating and, therefore, offer a more accessible form of support, allowing women to talk about their struggles naturally, without feeling like they were in a therapy session.

#### 3.2.5 Strengthening community-based support and engagement

##### 3.2.5.1 Bringing support closer to women's homes and familiar spaces

Service providers emphasised that mental health support must be physically and socially accessible to encourage engagement, particularly for women living in remote areas, facing financial difficulties, or balancing multiple caregiving responsibilities. Bringing services closer to where women lived or gathered was seen as a key solution to reducing barriers to care. Participants highlighted the value of offering mental health support in informal, community-based locations, such as community centres, libraries, schools, and local cafés. These environments were seen as more approachable and comfortable, reducing the fear or reluctance associated with seeking mental healthcare in formal clinical settings.

Another approach involved embedding mental health support into everyday activities that women were already engaged in, ensuring that accessing care did not feel like an added burden:

“Some women don't have the option to take time out for therapy or self-care in the way that we think of it. But if you bring therapy into something they're already doing, it makes a huge difference. They're still engaging in support, but in a way that doesn't add an extra task.” (P4, Healthcare professional)

Home visits were recommended as a means of overcoming barriers such as transportation difficulties, mobility issues, and childcare responsibilities, especially for women with multiple children who may find it difficult to leave their homes:

“Actually, home visits, I think, would be a huge step forward, having people in the home so that they don't have to have that barrier of trying to get out and about to access the services, especially with regards to, “I'm tipping up with my three babies and I can't take a pram in.” (P15, Charity worker)

In addition, home visits could help reduce stigma by allowing women to receive support in a private and familiar environment. These approaches enabled women facing practical and logistical barriers to receive the care they needed in a way that felt natural, convenient, and non-intimidating.

##### 3.2.5.2 Building trust through peer support and community engagement

Community engagement was consistently recognised as essential for building trust and improving access to PMH services. Service providers emphasised the importance of working closely with local organisations that have established connections within various communities, to design services that reflect the specific needs and priorities of the women they support. They highlighted the value of leveraging these existing networks and resources to ensure that services are better aligned with the needs of communities, making them more accessible and culturally relevant:

“We've found that by going out into the community and building relationships with local organisations, we're able to reach more women who may not have known about our services otherwise. We hold information sessions at community centres and work with trusted community leaders to spread the word.” (P8, Social enterprise worker)

Peer support groups were also identified as an effective way to create safe spaces where women could share experiences, discuss challenges in accessing care, and connect with others facing similar struggles. These groups provided women with a sense of belonging and reassurance, helping them feel heard and understood in ways that traditional services sometimes could not. One community worker explained the practical benefits of peer support:

“In our support groups, women can talk to each other and learn from each other's experiences. They share what they're going through, how they managed their situations and what worked for them. We can also refer them to a service or help them with self-referrals, from filling forms online to making calls or getting a contact number… it all depends on the individual and what they need.” (P7, Community worker)

For many women, these groups offered a space where they could speak openly without fear of judgment. One mother described how being part of a peer support group allowed her to feel understood:

“It's a supportive community where you can ask questions and get advice from other women who are also struggling with what you're going through. It helps you feel seen and heard, and you're understood as well because the mams, they know what you're talking about. It's incredibly helpful.” (P30, Mum)

For women facing particularly traumatic experiences, such as the loss of a child, peer support played a crucial role in helping them process grief and emotions. One participant explained how connecting with someone who had been through a similar experience helped her feel less isolated:

“When you lose a child, it's a very traumatic experience. I wasn't coping… wasn't coping at all. So I went to this group, and that's where I met (Name). She had been through the same experience last year, and she was describing all these feelings and I thought, oh my God! That's me right now. It was super helpful for me to know I wasn't alone and I could find this space where it was okay for me to not be okay, if that makes sense.” (P34, Mum)

In addition to peer support groups, service providers described the role of special interest groups in tailoring care to specific populations, such as LGBTQ + groups, ethnic minorities, and others facing additional barriers to accessing services.

Service providers noted that open discussions within these trusted spaces helped reduce stigma surrounding mental health by making it a less intimidating topic and promoting a more inclusive understanding of mental health. Participation in these groups also gave women opportunities to influence the design and delivery of services through involvement in community activities and advocacy efforts, ensuring that mental health support was both accessible and relevant to their lived experiences. These community-driven efforts were instrumental in providing pathways to support, helping to reduce the barriers that prevented women from seeking help or accessing PMH services.

#### 3.2.6 Integrating PMH support into other services supporting women

It was well recognised that women from disadvantaged backgrounds often require support across multiple areas, including housing, rehabilitation, domestic abuse services, and financial assistance. However, PMH support was often siloed from other essential services, leading to fragmented care and poor coordination. A lack of communication between services meant that seamless referrals to PMH support were not always possible, leaving many women without the help they needed. This fragmentation was particularly evident for those accessing social or charitable services, as frontline workers often lacked the necessary information to guide women toward PMH resources:

“We work with women who may be experiencing poverty, social isolation, or many other problems. But because everything is so disconnected and different, and the services don't really communicate with each other, it means that we may not be aware of the perinatal mental health support available to women in our community. And because of that, we can't also pass on this information to them, so they won't know about it.” (P12, Charity worker)

Some organisations recognised the significant mental health needs of the women they served–such as those experiencing homelessness–but lacked the mandate or resources to provide direct support. This lack of integration was particularly concerning in cases where mental health and other challenges, such as substance use, were deeply intertwined. Several providers highlighted that mental health services often excluded women struggling with substance use, despite the clear overlap between the two issues:

“I've been involved with the drug and alcohol services for so, so many years and, you can see how interlinked mental health is with substance use, but the mental health services won't on the whole touch people who are using substances because it's like, well you need to address that issue first, but then it's like, well, sure it's a mental health issue as well, so it's all kind of, what can they do? Because mental health services won't take you on.” (P15, Charity worker).

As a result, many women were unable to access PMH support until they had addressed their substance use – an often unrealistic expectation given the complex nature of these challenges.

Since women from disadvantaged backgrounds are more likely to have contact with social services, providers emphasised the need for stronger collaboration between social workers, health professionals, and PMH services to bridge care gaps. Some suggested that social services could serve as a key access point for PMH support, particularly for women receiving assistance during pregnancy or postpartum:

“Women in these (deprived) communities are more likely to interact with social services for various things that may be affecting their wellbeing, so if the social services can give them information about the types of services we have, how to access different types of support, even the importance of seeking help, that will go a long way.” (P7, Community worker)

However, service providers also described fear of social services involvement, particularly concerns about child custody, as an important barrier to engagement. This concern was especially pronounced among women experiencing multiple forms of disadvantage, including substance use, low income, homelessness and refugee status:

“We usually find that many families wouldn't want to have anything to do with us because we're social workers, and to them, I guess having a social worker means there's something wrong with your parenting, or some other issue, so no, they do not want to have a social worker.” (P6, Social worker)

Providers also described instances where fear or uncertainty about the role of social services led women to avoid contact with professionals altogether. A community health visitor described this in relation to Roma mothers:

“For example, we had these Roma mams that we were trying to reach, but each time we got to their house, they would not open the door for us. So, we started asking questions and spoke to some other people in that community, and that's when we got to understand that they thought we were social workers coming to take their kids away. And so, I think there is some uncertainty about what social workers do for families, and that fear can make them want to avoid every professional that comes to them.” (P13, Community health visitor)

These concerns could limit the role of social services as a route into PMH support by discouraging engagement with professionals who may otherwise identify, signpost or refer women for support. Providers therefore emphasised the importance of building trust and improving understanding of the role of social services.

Frontline workers in social services, housing support, and domestic abuse charities were often the first point of contact for women in distress, yet many lacked the knowledge to identify PMH difficulties or connect women with appropriate care. Providers advocated for training to help non-health professionals recognise PMH concerns and signpost women to relevant services:

“We don't expect housing officers or debt advisers to become mental health experts, but they could at least be able to spot the signs and know who to refer to. Sometimes, a simple question like “how are you coping?” can open up a whole conversation and lead to someone getting help they didn't even know existed.” (P21, Social worker)

Beyond training, providers emphasised the need for stronger referral pathways and co-located services to ensure women could access PMH support within the services they were already engaged with. Some suggested embedding mental health professionals within social services, housing agencies, and domestic abuse shelters to provide immediate support and reduce barriers to care. Others called for better coordination between PMH services and other sectors, with clearer communication and joint working agreements to prevent women from falling through the gaps.

## 4 Discussion

This study explored barriers and facilitators to accessing PMH services among women experiencing socioeconomic disadvantage in the NENC region. It included women with lived experience of PMH difficulties and service providers supporting them. Key findings highlighted gaps in routine mental health discussions, limited service visibility and signposting, the need for more flexible and culturally responsive support, the importance of community-based approaches, and better integration of PMH support with other services. Although identified within the NENC context, many of these findings are consistent with evidence from elsewhere in the UK, as discussed below.

Participants described limited discussion of mental health during routine antenatal care, with physical health often taking priority. This is consistent with previous studies showing that antenatal care often prioritises physical health over psychological wellbeing, leaving gaps in mental health discussions and screening (25, 26). NICE guidance recommends that women are asked about their emotional wellbeing at each routine antenatal contact (27). This lack of integration perpetuates missed opportunities for prevention, potentially delaying access to care until symptoms become more severe. Ensuring that mental health is embedded within routine maternity services requires a shift in professional training and service delivery, with clearer frameworks for discussing PMH proactively rather than reactively.

Limited visibility of PMH services, unclear signposting and difficulties navigating available support were recurring barriers, particularly for women unfamiliar with the healthcare system. This is consistent with research showing that service fragmentation, unclear signposting and inconsistent outreach can hinder engagement with healthcare services, especially among women from marginalised backgrounds (28–30). The assumption that individuals will proactively seek support fails to account for structural inequities such as digital exclusion, language barriers, and distrust of statutory services. Even as telehealth and digital interventions expand service reach, many women remain unaware of these options or struggle to navigate digital platforms (31, 32). Expanding proactive outreach, clearer signposting, and targeted engagement efforts is critical to ensuring both service visibility and accessibility.

Cultural and religious norms, stigma and perceptions of mental illness shaped how women understood mental health, whether they sought help, and how comfortable they felt engaging with PMH services. These findings reinforce the importance of cultural competence in equitable PMH care, particularly in light of wider ethnic inequalities in healthcare access and experience (30). Previous research has similarly shown that cultural norms influence mental health perceptions and help-seeking behaviours, with stigma and distrust acting as significant deterrents (33–37). While practitioner training is valuable, cultural competence must be embedded at an institutional level to ensure services are inclusive, accessible, and responsive to diverse needs. Without systemic changes, services risk remaining misaligned with the lived realities of many women, further limiting engagement and uptake.

Trusted community settings, peer support and relationships with local organisations were important in facilitating engagement with PMH support. This aligns with existing research showing that mistrust of formal services can deter help-seeking among marginalised groups (34–36). This study reinforces the need for a more transparent, community-led approach to service provision – one that prioritises collaboration over surveillance. Strengthening relationships between healthcare providers and communities through co-designed services, peer support models, and partnerships with grassroots organisations could help address longstanding barriers and promote trust in PMH services.

At a systemic level, the lack of integration between PMH services and other essential support systems reflects broader issues of care fragmentation, a challenge widely documented in maternal healthcare research (3, 38). Cross-sector collaboration is a crucial mechanism for improving continuity of care, yet many women continue to fall through the gaps due to poor coordination and restrictive service eligibility criteria (3). Structural reforms that embed mental health within existing community-based services and strengthen referral pathways are essential to ensuring coordinated rather than fragmented care (7, 38). However, these reforms cannot be effectively implemented without addressing system-wide constraints, including workforce shortages, high caseloads, and service pressures. While these challenges are recognised in the literature (39) and acknowledged by service providers in this study, they were not the primary focus in this paper. Under-resourced maternity and mental health services result in insufficient time for meaningful mental health discussions in antenatal care, prolonged waiting times for specialist support, and increasing reliance on overstretched voluntary sector organisations to fill gaps (3, 40, 41).

### 4.1 Recommendations

These findings reinforce the need for policy solutions that prioritise investment in the maternal mental health workforce, improve training for frontline professionals, and secure sustainable funding for community-based support. Without addressing these structural limitations, efforts to enhance service accessibility and integration will remain constrained.

To address barriers to PMH support, a multi-level approach is required to improve both the availability and accessibility of PMH services. First, systematic integration of mental health into routine maternity care should be prioritised, with clear guidelines and training for healthcare professionals to facilitate proactive discussions. Second, improving service visibility through targeted outreach, clear signposting, and multilingual information campaigns is essential to ensuring that women are aware of and can navigate available support. Third, cultural competence must be embedded at both practitioner and institutional levels, ensuring that PMH services are designed with the needs of diverse communities in mind. This includes recruiting a diverse workforce, adapting services to reflect different cultural perspectives on mental health, and engaging with trusted community leaders to build stronger relationships between healthcare providers and service users.

Additionally, strengthening trust in statutory services requires a shift toward collaborative, community-led models of care delivery, with co-designed services and peer support initiatives playing a central role. Finally, addressing workforce shortages and system pressures is critical for improving access and quality of care. Sustainable funding for PMH services, increased investment in the maternal mental health workforce, and policies that support cross-sector collaboration are necessary to reduce waiting times, enhance service coordination, and ensure that all women, regardless of socioeconomic or cultural background, can access timely, high-quality perinatal mental health support.

### 4.2 Strengths and limitations

This study has notable strengths. Capturing perspectives from both women with lived experience and service providers supporting them provides a well-rounded understanding of access barriers and facilitators. Engaging women from diverse backgrounds and socioeconomically disadvantaged groups further enhances the study's relevance. However, some groups, such as women experiencing homelessness, sex workers, and lesbian or transgender individuals, were not directly represented, though insights from service providers working with them helped address this gap. Future research should prioritise direct engagement with these underrepresented groups to strengthen the evidence base for improving PMH service accessibility.

## 5 Conclusion

This study highlights the need for fundamental improvements in PMH service accessibility. While barriers to care are well-documented, efforts to improve access must go beyond availability to ensure services are visible, culturally responsive, and trusted. Addressing these challenges requires sustained investment in workforce capacity, service integration, and proactive community engagement. Future research should prioritise direct engagement with underrepresented groups to further inform inclusive and effective service improvements.

## Data Availability

The raw data supporting the conclusions of this article are not publicly available due to the sensitive nature of the data and the need to protect participant confidentiality. Requests for access to anonymised data will be considered where relevant, subject to appropriate ethical and governance approvals.
