# Supplementary material for: Bridging gaps in perinatal mental health services for socioeconomically disadvantaged women
Source: Front Glob Womens Health. 2026 Sep 23;7:1850520. doi: 10.3389/fgwh.2026.1850520 (PMC13642319; doi:10.3389/fgwh.2026.1850520)
Supplement: Supplementary file 1 [file Table1.docx]

**Supplementary Table 1. Sociodemographic characteristics of Phase 2 participants (n=20)**

| **Characteristic** | **Category** | **n** |
| --- | --- | --- |
| **Age** | <20 years | 3 |
|  | 25-35 years | 14 |
|  | >35 years | 3 |
| **Marital status** | Married | 9 |
|  | Divorced | 4 |
|  | Single | 7 |
| **Ethnic background** | White | 9 |
|  | Asian / Asian British | 3 |
|  | Black / Black British / Caribbean / African | 6 |
|  | Mixed | 2 |
| **Employment status** | Employed | 16 |
|  | Not employed | 4 |
| **Number of children** | 1 | 6 |
|  | 2 | 5 |
|  | ≥3 | 9 |
| **Age of youngest child** | <1 year | 12 |
|  | 1-2 years | 8 |

**Note.** Participants experienced multiple and overlapping forms of disadvantage, including financial/deprivation-related circumstances; single parenthood, childcare and wider caring pressures; limited social support; language and healthcare-navigation barriers; disability; domestic or sexual abuse; bereavement following baby loss; and culturally shaped barriers to help-seeking.
